# BdBG: a bucket-based method for compressing genome sequencing data with dynamic de Bruijn graphs

**DOI:** 10.7717/peerj.5611

**Published:** 2018-10-19

**Authors:** Rongjie Wang, Junyi Li, Yang Bai, Tianyi Zang, Yadong Wang

**Affiliations:** 1 School of Computer Science and Technology, Harbin Institute of Technology, Harbin, HeiLongJiang, China; 2 School of Computer Science and Technology, Harbin Institute of Technology (Shenzhen), Shenzhen, Guangdong, China

**Keywords:** Compression, Bucket-based, Next-generation sequencing, Dynamic de Bruijn graph

## Abstract

Dramatic increases in data produced by next-generation sequencing (NGS) technologies demand data compression tools for saving storage space. However, effective and efficient data compression for genome sequencing data has remained an unresolved challenge in NGS data studies. In this paper, we propose a novel alignment-free and reference-free compression method, BdBG, which is the first to compress genome sequencing data with dynamic de Bruijn graphs based on the data after bucketing. Compared with existing de Bruijn graph methods, BdBG only stored a list of bucket indexes and bifurcations for the raw read sequences, and this feature can effectively reduce storage space. Experimental results on several genome sequencing datasets show the effectiveness of BdBG over three state-of-the-art methods. BdBG is written in python and it is an open source software distributed under the MIT license, available for download at https://github.com/rongjiewang/BdBG.

## Introduction

The rapid development of sequencing technologies has made genome sequencing more affordable. Next-generation sequencing (NGS), also known as high-throughput sequencing, was introduced to the sequencing market in 2007. NGS enables sequencing with higher throughput and has drastically reduced the cost per genome sequencing (Loh, Baym & Berger, 2012). As a result, the amount of genome sequencing data has grown exponentially over the past decade, posing significant challenges to data storage. Raw sequencing data referred to as reads are stored in an ASCII-based text file in FASTQ format. Each read in the file has three main fields: (i) read identifier, (ii) read sequence and (iii) read quality score. To reduce storage and transmission costs, FASTQ files are often compressed with common compression tools such as gzip (http://www.gzip.org/) and bzip (http://www.bzip.org/). Although these traditional tools are fast and widely accepted, they are not optimal for genome sequencing data compression.

NGS data compression methods can be divided into two main categories: *reference-based* and *de novo* methods. Reference-based methods (Fritz et al., 2011; Kingsford & Patro, 2015; Zhang et al., 2015a; Zhang et al., 2015b; Arram et al., 2016; Huang et al., 2017) align the reads to the reference genome and record only the mapped positions and different nucleotides in the alignment instead of recording the entire read sequence. Although PATHENC (Kingsford & Patro, 2015) does not use the mapping strategy, it exploits the reference genome by encoding the nucleotide based on the previous k nucleotides with an arithmetic encoder (Solomon & Motta, 2010), if the next nucleotide appears in the reference genome, then it increases the coding probability of the nucleotide. Despite achieving high compression rates, these reference-based methods require considerable time for aligning reads to the reference genome. Moreover, during the compression and decompression, all of them require a reference genome. To eliminate the limitation of the reference genome requirements during decompression, Quark (Sarkar & Patro, 2017) encodes the reads in a semi-reference-based manner: the reference genome is required only for compression rather than decompression. However, when there is no reference genome, neither the reference-based nor semi-reference-based approaches are applicable.

The de novo methods exploit the coverage redundancy in NGS data. These methods can be roughly further divided into three types: context modeling methods (such as FQZCOMP (Bonfield & Mahoney, 2013) and DSRC2 (Roguski & Deorowicz, 2014)), reference constructing methods (such as QUIP (Jones et al., 2012), LEON (Benoit et al., 2015) and DARRC (Holley et al., 2018)), and read reordering methods (such as SCALCE (Hach et al., 2012), BEETL (Cox et al., 2012), MINCE (Patro & Kingsford, 2015), ORCOM (Grabowski, Deorowicz & Roguski, 2015), HARC (Chandak, Tatwawadi & Weissman, 2017), Assembltrie (Ginart et al., 2018) and FaStore (Roguski et al., 2018)).

The context modeling methods FQZCOMP (Bonfield & Mahoney, 2013) and DSRC2 (Roguski & Deorowicz, 2014) encode nucleotides based on an order-k model, using the previous k nucleotides to predict the next nucleotide, followed by the arithmetic encoder.

The reference constructing methods QUIP (Jones et al., 2012), LEON (Benoit et al., 2015) and DARRC (Holley et al., 2018) compress the reads by constructing a reference genome. QUIP utilizes partial reads to assemble the reference contigs. LEON uses all reads to build a de Bruijn graph (Compeau, Pevzner & Tesler, 2011) and encodes each read as a path in the de Bruijn graph. DARRC constructs a de Bruijn graph with spanning super reads that have been assembled by shared overlap reads. All of these reference constructing methods require storage of reference contigs or de Bruijn graph, which consume a non-negligible portion of the compressed file space.

The read reordering methods try to distribute the same or similar reads from the same locus or repetitive sequences as closely as possible. This step reduces the density of the read sequence entropy and contributes to downstream compression. Note that although the read order has changed, the reads produced by the NGS protocols are generated from random locations across the entire genome. In addition, these read reordering methods assume that the original order of reads does not represent any meaningful information.

SCALCE (Hach et al., 2012) applies Local Consistent Parsing (LCP) (Sahinalp & Vishkin, 1996) to generate core substrings for clustering reads into different bins and then encoded via 2/8 encoding, followed by gzip compression. BEETL (Cox et al., 2012) employs the Burrows-Wheeler-Transform (BWT) (Burrows & Wheeler, 1994) to group similar reads, after that, the reordered reads by BWT can be compressed via general compression techniques, such as run length encoding (Bassiouni, 1985) or Huffman encoding (Huffman, 1952).

ORCOM (Grabowski, Deorowicz & Roguski, 2015) utilizes the concept of ‘minimizer’ (Roberts et al., 2004) to dispatch similar read sequences into the same disk bin and compresses each bin separately. The minimizer is defined as the lexicographically smallest k-mer generated from the read sequence. The underlying intuition is that two similar read sequences might have a large probability of sharing the same minimizer. The read sequences in each bin are compressed independently by the context-based compressor PPMd (Shkarin, 2002) or the arithmetic encoder.

MINCE (Patro & Kingsford, 2015) first distributes the read sequences into the bucket that has the most shared k-mers. At the same time, to improve the speed of comparison, each read sequence is compared only to the buckets indexed by the k-mers generated from the read sequence itself. After that, it compresses the sequences in each bucket via PLzip  (https://www.nongnu.org/lzip/plzip.html), which is a lossless data compressor based on the Lempel–Ziv-Markov chain algorithm (LZMA).

HARC (Chandak, Tatwawadi & Weissman, 2017) reorders reads approximately according to the matching between the suffix of the current read and the prefix of the target read based on two hash tables, then HARC encodes them to remove the redundancy between consecutive reads. Assembltrie (Ginart et al., 2018) greedily constructs a cycle-rooted trie with each read as a node. The parent and children are the reads sharing the longest prefix and suffix overlap with the node based on k-mer hash tables, and each read is encoded with the difference from its parent. FaStore (Roguski et al., 2018) employs the ‘minimizer’ method to cluster reads into bins, assembles the reads within each bin into some possible large contigs, and finally encode the reads only with positions and variants in the contigs.

In the above reference constructing studies, the constructed reference sequence must be stored in a compressed file for decompression. However, the constructed reference sequence also consumes a considerable amount of space, which substantially affects the compression efficiency. In this study, we attempt to avoid storing the reference sequence while preserving the benefits of read reference constructing and reordering methods.

In this paper, we propose a novel compression method, BdBG, as a new compressor for genome sequencing data. BdBG consists of two main steps. First, similar reads are dispatched to the same bucket based on the highest score of shared k-mers. Second, the already encoded reads are used to construct a de Bruijn graph within each bucket separately, and each read is encoded as a list of bifurcations in the graph. To avoid storing the de Bruijn graph itself, we construct the de Bruijn graphs dynamically during encoding/decoding. We applied our method, BdBG, to eight different genome and transcriptome sequencing datasets. The results showed that the compression performance of BdBG is better than that of GZIP, LEON (Benoit et al., 2015) and MINCE (Patro & Kingsford, 2015), with improvements of up to 83%, 81%, and 52%, respectively.

## Materials & Methods

### Overview

**Figure 1:**
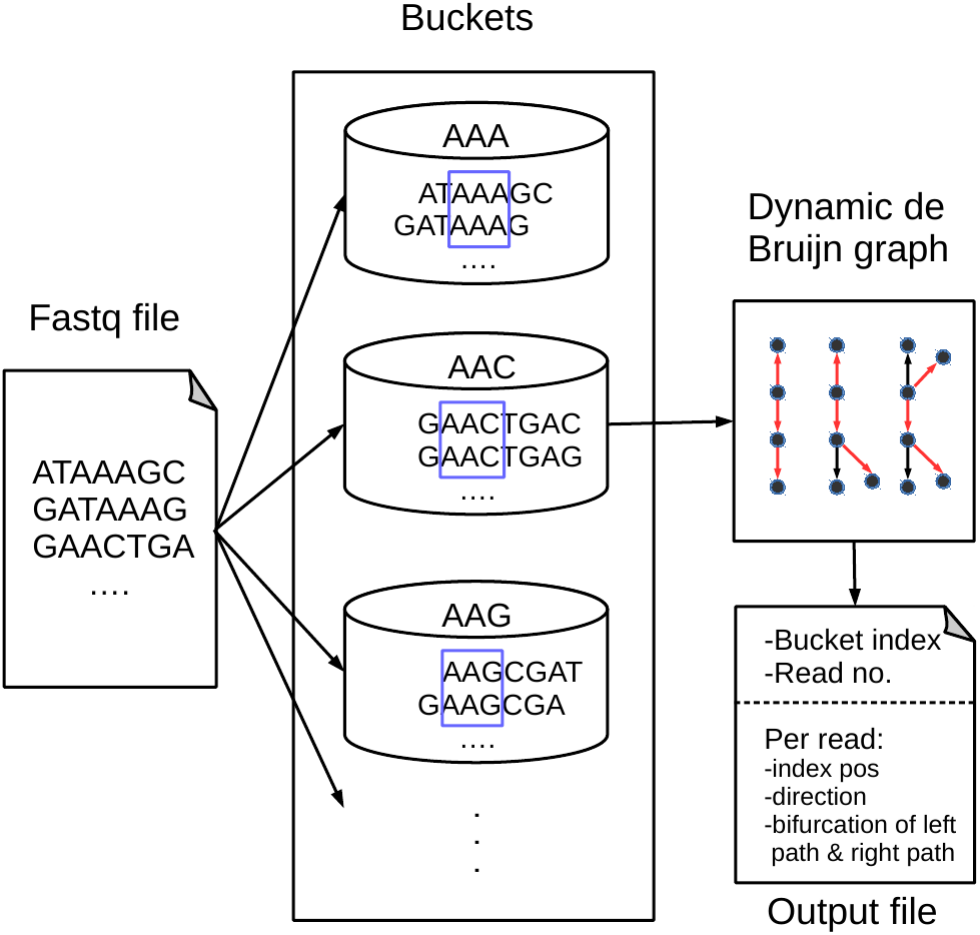
An overview of the BdBG method. First, assign similar reads to the same bucket based on the scores of shared k-mers between the read and k-mers in the bucket, and then each read is encoded as a path in a dynamic de Bruijn graph, which is constructed by the already encoded reads. The necessary information required to recover reads from the de Bruijn graph is stored in compressed files, including the bucket index, number of reads in the bucket, read index positions, directions of reads and a list of bifurcations for the left and right paths.

Figure 1 shows an overview of our proposed BdBG method, which includes read bucketing and compressing steps. Each step can be further divided into several small parts. The first step can be perceived as the MINCE (Patro & Kingsford, 2015) bucketing method: similar reads are dispatched into the same bucket based on the score of shared k-mers. The purpose of this step is to facilitate downstream compression. In the subsequent read compression step, a de Bruijn graph is constructed with reads already encoded as the reference sequence for encoding the read. Each read is encoded as a path in the de Bruijn graph. To avoid storing the de Bruijn graph, the de Bruijn graph is dynamically constructed with reads that have been encoded/decoded during compression/decompression. Finally, all the necessary output streams needed to recover the reads are compressed by a general compressor PLzip (https://www.nongnu.org/lzip/plzip.html).

### Read bucketing

Each bucket has a unique index label ℓ(*b*) and a collection of reads. When processing a read *r*, all $2( \left\vert r \right\vert -k+1)$ bucket indexes labeled with k-mers generated from *r* are checked; the factor of 2 is derived from considering both *r* and its reverse complement *rc*(*r*). By default, *k* = 15. The read *r* is assigned to the bucket have the highest score $\bar {b}$ that satisfies

(1) \begin{eqnarray*}\bar {b}={argmax}_{b\in Z(r,k)}\{Z(r,k) \cap {\Sigma }_{{r}^{{^{\prime}}}\in bucket(b)}Z({r}^{{^{\prime}}},k)\}\end{eqnarray*}

where *Z*(*r*, *k*) denotes the set of all k-mers in the read *r* and Σ_*r*′∈*bucket*(*b*)_*Z*(*r*′, *k*) denotes all k-mers generated from the reads in the bucket *b*. The similarity between read *r* and bucket *b* is defined as the number of shared k-mers. The read *r* is dispatched to the bucket with the highest score.

If the highest score bucket $\bar {b}$ from Eq. (1) is indexed by the k-mer generated from the forward direction of read *r*, then the read *r* is allocated to the bucket; otherwise, if bucket $\bar {b}$ is indexed by the k-mer generated from the reverse complement of read *r*, then *rc*(*r*) is allocated to the bucket. If no bucket is indexed with the k-mer generated from the read *r* or *rc*(*r*), a new bucket is created. Initially, the new bucket is indexed with a minimizer (Roberts et al., 2004), which is defined as the lexicographically smallest k-mer in *r* and *rc*(*r*).

The reads in each bucket are sorted according to the bucket index positions and nucleotides, which further brings similar sequences close to each other and has a positive impact on subsequent compression.

#### Singleton reassigning

In an extreme case, a bucket may contain only one read after bucketing all reads. This is called a singleton bucket. The read in the singleton bucket is hard to compress due to the lack of a reference. To eliminate the singleton bucket effect, we adopt the same ‘rescue’ procedure as MINCE (Patro & Kingsford, 2015): when all read allocations are completed, all singleton buckets are deleted, and the reads in these buckets are reassigned to the remaining non-empty buckets according to Eq. (1). In the new reassignment process, reads are more likely to be dispatched to other non-empty buckets, because there are more buckets available for allocation. If there are still reads that cannot be allocated to any non-empty buckets, they are directly dispatched into a special bucket indexed by an empty string, and finally compressed into the beginning of the output stream file in a 2-bit (*A* = 00, *C* = 01, *G* = 10, *T* = 11) manner.

#### Read bucketing output

The outputs of read bucketing include the bucket description files: *f*_*index*_ and *f*_*cov*_, and a series of read description files: *f*_*indexPos*_, *f*_*rc*_, *f*_*len*_, *f*_*N*_ and *f*_*order*_. The contents of these files have the following meaning:

• *f*_*index*_—a list of bucket indexes, each of which is a common subsequence of reads within the bucket and serves as the starting node for each read path in the following de Bruijn graph. The bucket indexes are stored in ascending order, transformed into the 2-bit manner and compressed with delta encoding.
• *f*_*cov*_—the number of reads in each bucket. For the number of reads in each bucket has a wide range, each value is encoded in 32-bit.
• *f*_*indexPos*_—a list of the positions of the bucket index in the reads. Since the NGS read data have a relatively short length, each position is sorted in ascending order and encoded with 8-bit and compressed with delta encoding.
• *f*_*rc*_—a list of binary data in which a ‘0′ indicates that the read in a forward direction and a ‘1′ indicates the read in a reverse-complement direction.
• *f*_*len*_—the length of each read. Each length is encoded in 8-bit, and it is necessary to restore the unequal length of read sequences.
• *f*_*N*_—the position and length of the character “N” in the raw read sequences. Both pieces of information are encoded in 8-bit. In the raw read sequences, all character “N” is replaced by nucleotide “C”.
• *f*_*order*_—the orders of reads in the raw file. Each order is encoded in 32-bit. For the single-end reads, the output of this file is optional. For the paired-end reads, this information is enforced to restore the paired-end information correctly.

In the end, all of these output files are compressed separately using the PLzip compressor as part of the final output stream.

### The de Bruijn graph

**Figure 2:**
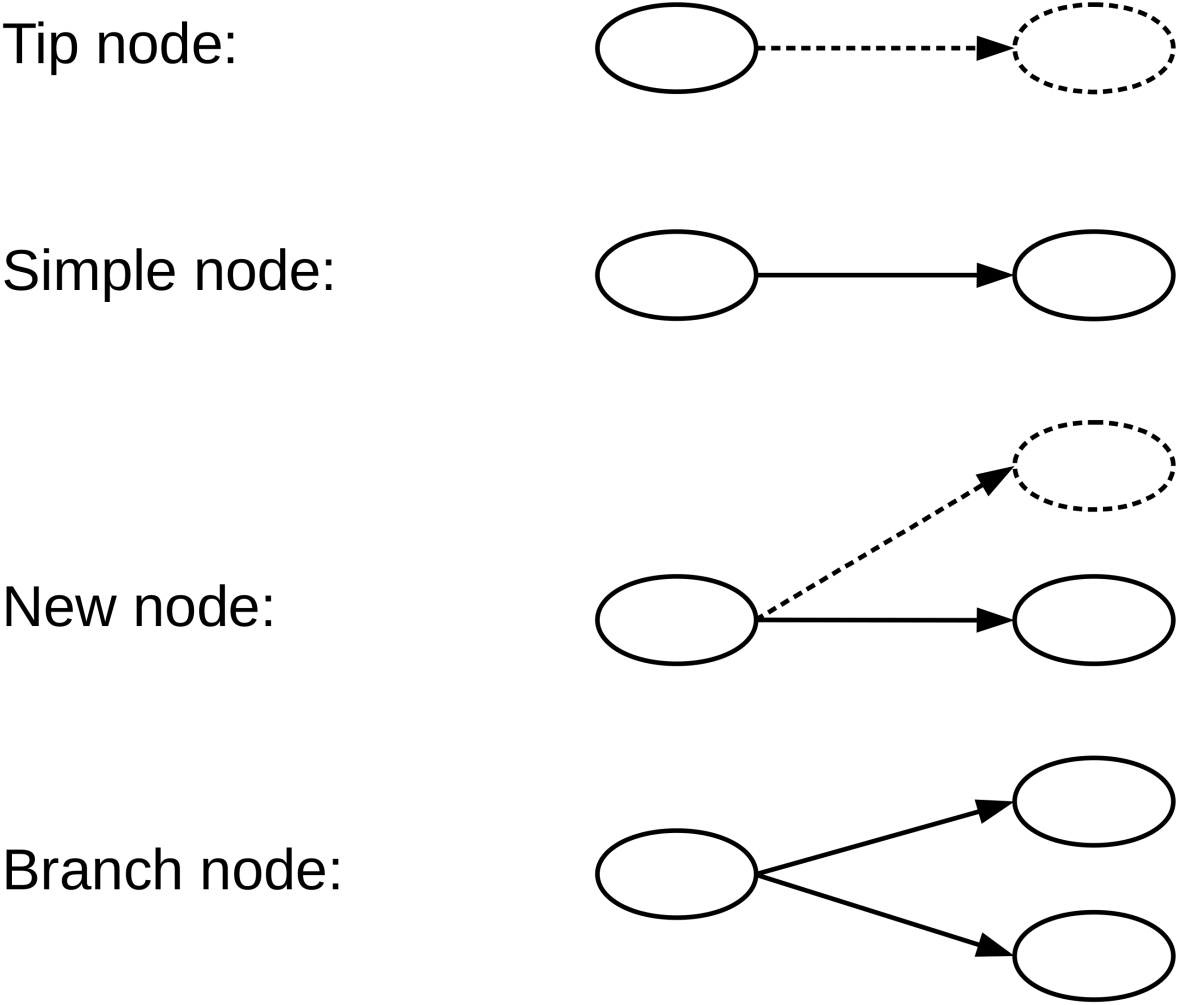
We define four types of nodes in the de Bruijn graph. A Tip node is a vertex without any successor. A Simple node is a vertex with only one successor, and its successor is identical to the read next nucleotide. A New node is a vertex with only one successor, but the successor is different from the read next nucleotide. A Branch node is a vertex with more than one successor. The dotted lines indicate that the read nucleotide corresponding node does not exist in the current graph.

A de Bruijn graph (dBG) is a directed graph G = (V, E) consisting of a set of vertices V and a set of direct edges E. A vertex *v* ∈ *V* represents a distinct k-mer generated from the reads, and a directed edge *e* ∈ *E* represents a (k-1)-length overlap between the suffix *v* ∈ *V* and prefix *v*′ ∈ *V*. Each vertex has $ \left\vert \mathcal{A} \right\vert $ possible successors and $ \left\vert \mathcal{A} \right\vert $ possible predecessors, for the genome sequencing data $\mathcal{A}=\{A,C,G,T\}$. We define the type of a given vertex according to the number of successors and whether the successor is equal to the encoded read path. Four types of nodes that we have defined in the de Bruijn graph, namely, Tip nodes, Simple nodes, New nodes, and Branch nodes, as shown in Fig. 2. A Tip node is a vertex without any successor. A Simple node is a vertex with only one successor and its successor is identical to the read path. A New node is a vertex with only one successor node, but the successor node is different from the read path. A Branch node is a vertex with multiple successors.

Once a de Bruijn graph has been constructed from a set of reads *R* with a k-mer size k, any read *r* ∈ *R* can be represented as a path of the graph containing $ \left\vert r \right\vert -k+1$ nodes (Limasset et al., 2016). To encode the read sequences, we only need to record the path $ \left\vert r \right\vert -k+1$ nodes and starting node information, which can be used to recover all read sequences from the de Bruijn graph in a lossless way.

There are only four types of nodes in the graph as we define, and we record different information for different node types. If it is a Simple node, nothing needs to be recorded; if it is a New node, the position and nucleotide need to be recorded; if it is a Tip node or Branch node, the read nucleotide needs to be recorded. We assume that the majority of the read path is composed of Simple nodes and that only a small part of the path is composed of the other three type nodes. This allows the read to be compressed effectively, and in fact, the read sequences conform to this feature because it has a high degree of repeatability and consistency.

However, in order to reconstruct the read sequence with path information, the de Bruijn graph needs to be stored, and the graph itself also takes up a non-negligible storage space. To avoid storing the de Bruijn graph and to improve the compression efficiency, we propose a dynamic de Bruijn graph method.

#### Dynamic de Bruijn graph

When encoding/decoding a read, we construct a de Bruijn graph as the reference for this read using all reads that have already been encoded/decoded in the bucket. Since the de Bruijn graph is constructed dynamically from read by read after encoding/decoding and synchronously constructed on both the encoder and decoder sides from an empty graph, there is no need to store any information of the de Bruijn graph.

The de Bruijn graph is initialized with an empty graph when the first read sequence is encoded/decoded, the path nodes are the Tip nodes for this sequence. Therefore, for the Tip nodes, all nucleotides need to be stored, all of the first read sequences for each bucket is saved into a file *f*_*firSeq*_ and encoded them in a 2-bit manner.

#### Encoding the read sequences

We encode each read as a path of the de Bruijn graph, and the path is described with a start position and a list of bifurcations in the graph. The start position, as the read path ‘anchor’, is the bucket index that shared with all reads in the bucket. From the ‘anchor’ position, the path is separated into a left and a right path.

When the read from the ‘anchor’ node is encoded in any direction, there are four possible nucleotide successors {A, C, G, T} in the de Bruijn graph. To avoid excessive Branch nodes due to sequencing errors and mutations, we filter successor node abundance ratios less than a fixed proportion threshold (0.2 by default). According to the number of successors and the following nucleotide in the read, the node has been classified into four different types, as defined in Fig. 2. For different types of nodes, we encode the read path information into the bifurcation file *f*_*bifur*_ in different ways.

If the node is a Simple node, nothing needs to be encoded. If it is a Tip node, the read nucleotide is encoded in the 2-bit manner to the file *f*_*bifur*_. If it is a Branch node, the nucleotide in the path is encoded into file *f*_*bifur*_ with the arithmetic encoder. The encoding probability used by the arithmetic encoder is obtained from the frequency distribution of the successors. In the worst case, if it is a New node, the New node position and read nucleotide are encoded into the bifurcation file *f*_*bifur*_. Since the nucleotide of the read must be different from the only one successor nucleotide of the New node in the graph, the nucleotide we want encoding is one of three remaining nucleotides. According to the entropy of information theory (Shannon, 1948), only approximately log_2_3 bits are needed to encode this nucleotide with the arithmetic encoder.

**Figure 3:**
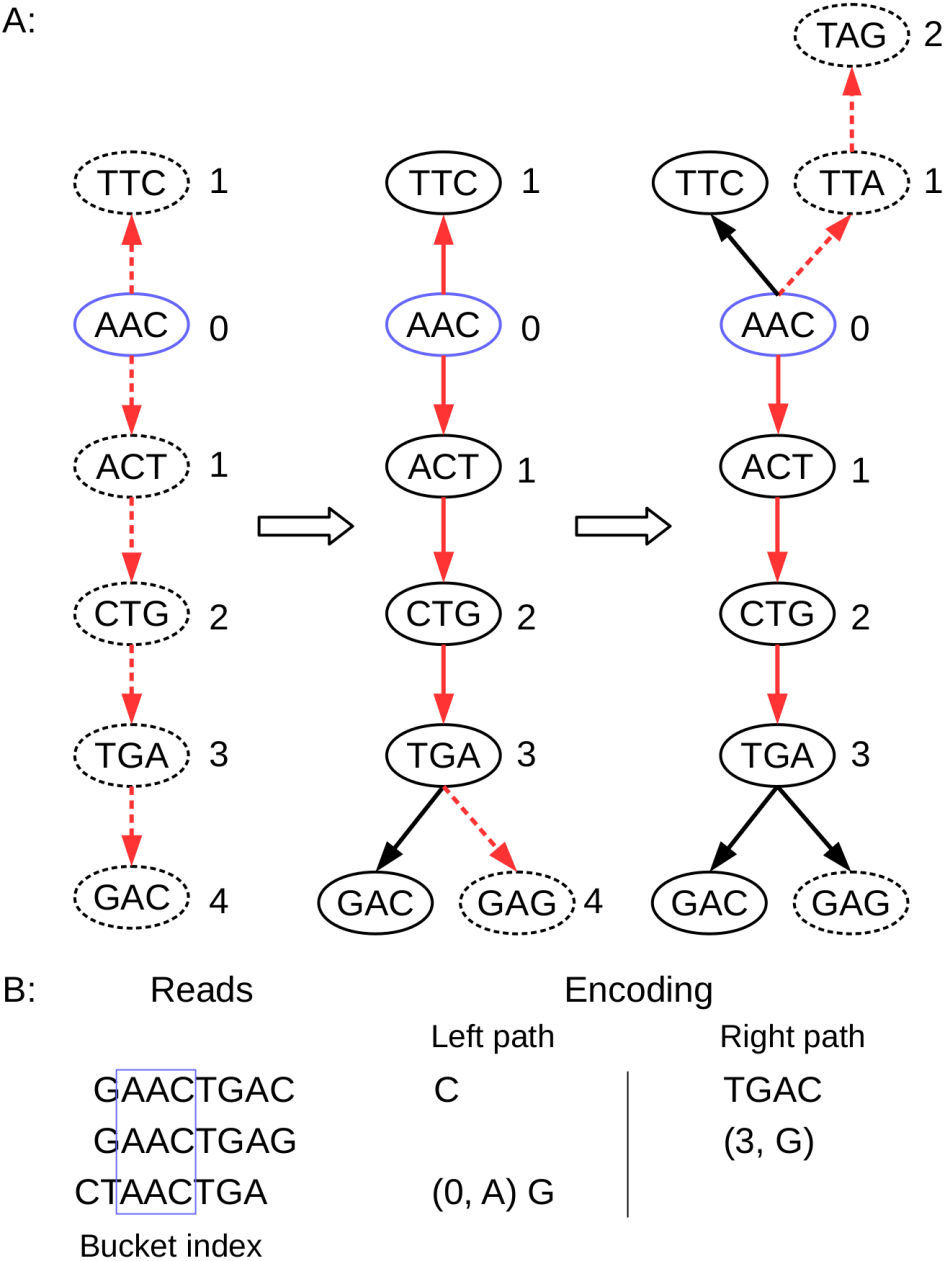
Schematic description of BdBG path encoding. (A) the path of three reads in the de Bruijn graph is depicted, where the k-mer size is 3. The index anchor node is shown in blue, and the left path and right path from the anchor are highlighted in red. The dotted lines indicate that the read path nodes do not exist in the current de Bruijn graph. (B) the corresponding reads and the left path (in reverse complement, A ↔ T, C ↔ G) and the right path encoding list for these three reads are shown. Note that the first read requires the information for all nodes (initialize graph = ∅); the second read only need to record a New node (3, G); and the third read needs to record a New node (0, A), and a Tip node (G).

When the nucleotide encoding is complete, the current node moves to the next node on the read path and continues the encoding process described above, until the entire read sequence is encoded. The details of the encoding procedure are shown in Algorithm 1, and an encoding example is shown in Fig. 3.

 
_______________________________________________________________________________ 
 Algorithm 1: The pseudocode of encoding one read with the dynamic de 
  Bruijn graph in the BdBG method. 
________________________________________________________________________________ 
    Input  : One read R in a bucket, read index position α. 
    Output: Bifurcation left path list Lp and right path list Rp for read R. 
 1  // encode the right path 
 2  k–mer ← R[α,α + k − 1]; 
 3  for i ← α to |R|− k + 1 do 
 4    Rsucc ← Successors_in_graph(k–mer); 
 5    if size of Rsucc = 1 then 
 6      if R[i + k] = Rsucc then 
 7        // Simple node, nothing to do. 
 8      else 
 9        // New node, add read nucleotide and position to the path. 
10        Rp.add(R[i + k],i + k); 
11      end 
12      k–mer ← Rsucc; 
13    else 
14      // Tip node or Branch node, add read nucleotide to the path. 
15      Rp.add(R[i + k]); 
16      k–mer ← Suffix(k–mer,k − 1) + R[i + k]; 
17    end 
18  end 
19  // encode the left path 
20  k–mer ← rc(R[α,α + k − 1]); 
21  for i ← α to 0 do 
22    Lsucc ← Successors_in_graph(k–mer); 
23    if size of Lsucc = 1 then 
24      if rc(R[i + k]) = Lsucc then 
25        // Simple node, nothing to do. 
26      else 
27         // New node, add read nucleotide and position to the path. 
28         Lp.add(rc(R[i + k]),i + k); 
29      end 
30        k–mer ← Lsucc; 
31    else 
32      // Tip node or Branch node, add read nucleotide to the path. 
33      Lp.add(rc(R[i + k])); 
34      k–mer ← Suffix(k–mer,k − 1) + rc(R[i + k]); 
35    end 
36  end 
37  // add all k–mers generated by read R into the de Bruijn graph. 
38  graph.insert(ℤ(R,k)); 
39  return Lp,Rp; 
____________________________________________________________________________________     

To efficiently store the nucleotides, we store the nucleotides and the positions separately from the bifurcation file. We add a binary New nodes flag file *f*_*numFlag*_ to indicate whether the current node is a New node. If the node is a New node, we add a bit ‘1′ to the file *f*_*numFlag*_; otherwise, if the node is a Tip node or a Branch node, we add a bit ‘0′ to the file *f*_*numFlag*_.

When encoding the left path of the read, we use the reverse-complement of the read, so that the process of encoding the left path is exactly the same as the right path. After encoding the read *r*, we insert all k-mers generated from *r* to the de Bruijn graph as the following reads reference.

####  Handling paired-end reads

The sequencing data can be generated from one or both ends of a fragment, which is known as a single-end or paired-end library type. The latter type is widely used in many areas of sequence analysis, such as genome assembly, read error correction and variant calling. The paired information is in the form of two reads residing on the same lines in two files or alternatively located in a single file. BdBG stores the initial orders of reads to avoid the loss of paired information caused by read bucketing. Because the original orders of reads have a random uniform distribution after bucket rearrangement, storing the entire order information requires approximately *N*∗*log*_2_(*N*) bits, where *N* is the number of reads.

#### Decompression

The main difference between the decompression and compression processes is that the decompression process does not require read bucketing process. The decompression process first decodes the first read sequence of each bucket from the file *f*_*firSeq*_, the bucket index from the file *f*_*index*_, and the number of reads per bucket from the file *f*_*cov*_. The length and bucket index position per read are decoded from the file *f*_*len*_ and the file *f*_*indexPos*_, so the length of the left and right paths per read in the de Bruijn graph is fixed.

We take decoding the right path as an example (the left path is symmetrical; the only difference is that the decoded sequence is in reverse-complementary). We decode the beginning of read using the bucket index as the read ‘anchor’, and four possible nucleotide successors are queried in the de Bruijn graph. The number of successors for the node and the auxiliary information from the file *f*_*numFlag*_ are queried to determine the node type. The node can only be one of the four types of nodes defined in the encoding section. For different node types, we use different decoding strategies for the read nucleotide.

If no successor exists, the current node is a Tip node, and we take 2 bits of data from the file *f*_*bifurR*_ and decode the nucleotide in the 2-bit manner.

If there is only one successor, the next 1 bit of data from the file *f*_*numFlag*_ is queried to assist determine the node type. If it is true, the node is a New node, and log_2_3 bits of data from the file *f*_*bifurR*_ are loaded and decoded it with the arithmetic decoder. Since we already know that the only successor is not the read nucleotide *X*, for *X* ∈ {*A*, *C*, *G*, *T*}, we only need to decode the nucleotide in the three remaining nucleotides {*A*, *C*, *G*, *T*}∖{*X*}. If it is false, the node is a Simple node, and thus, the successor nucleotide is exactly the read nucleotide we want to decode.

If more than one successor exists, the node is a Branch node, the probability distribution of the successors is queried from the de Bruijn graph, and the nucleotide is decoded by the arithmetic decoder from the file *f*_*bifurR*_.

After decoding the read nucleotide, the current node moves to the next node in the read path and continues the above decoding process until the entire left and right paths are decoded. After that, 1 bit from the file *f*_*rc*_ is loaded to determine whether the read should be converted to the inverse complementary sequence. Then, all k-mers generated from the read are inserted into the de Bruijn graph as the reference sequence for the subsequent read decoding. Finally, the read characters “N” information is recovered from the file *f*_*N*_.

To decode paired-end read sequences, we rearrange the read orders according to the information in the file *f*_*order*_. Thus, the output reads are the same as the original reads, and the paired-end information is preserved.

#### Complexity

BdBG executes compression in two passes over reads: one pass dispatches similar reads into the same bucket, and another pass encodes the read sequence into a list of bifurcations based on the de Bruijn graph. The latter is the only pass in the decompression process. For a given read sequence, dispatching it into a bucket and encoding it in the de Bruijn graph both require a number of operations proportional to the number of k-mers in the read sequence itself. Thus, the time complexity for compression/decompression is *O*((*L* − *k* + 1)∗*N*) = *O*(*N*), where L is the read length, k is the k-mer size and N is the number of read sequences.

In both the compression and decompression processes, two main data structures are stored in the main memory: the raw read sequences and the k-mers generated from the read sequences. These data structures consume a maximum space and complexity equal to *O*(2*LN* + 2*k*(*L* − *k* + 1)*N*) = *O*(*N*) in the compression/decompression process, where L is the read length, k is the k-mer size and N is the number of read sequences, 2 is the fact that each nucleotide takes up 2 bits of space.

## Results

We performed experiments with our BdBG method on eight different real genome and transcriptome sequencing datasets. All datasets were obtained from the National Center for Biotechnology Information (NCBI) Sequence Read Archive (SRA) database and can be downloaded according to the dataset ID, as described in Table 1. These datasets were generated from different library types, organisms, and Illumina technologies. Since our BdBG compression method did not preserve the read identifiers and the quality scores in the FASTQ file, we only compared the compression results of the read sequences in the FASTQ file.

**Table 1:** Characteristics of the datasets used in the experiments.

Dataset ID	Library type	Organism	Technology	Read no.	Len
ERR1147042	PE	C. albicans	Illumina GA IIX	2,969,502	101/101
ERR034088	PE	S. enterica	Illumina GA II	1,728,241	60/55
SRR554369	PE	P. aeruginosa	Illumina GA IIX	1,657,871	100/100
SRR959239	PE	E. coli str.K-12	Illumina HiSeq 2000	2,686,416	98/98
ERR418881	PE	S. aureus	Illumina GA IIX	1,785,385	108/108
MH0001.081026	PE	H.sapiens Gut	Illumina GA	11,640,674	44/44
SRR327342	PE	S.cerevisiae	Illumina GA	15,036,699	63/75
SRR037452	SE	H.sapiens	Illumina GA	11,712,885	35

We compared our proposed BdBG method against three compression methods: GZIP (http://www.gzip.org/), LEON (Benoit et al., 2015), and MINCE (Patro & Kingsford, 2015). The last one, MINCE was among the top de novo compression tools in the recent survey (Numanagić et al., 2016). The compression results are shown in Table 2; default parameters were used for all compression tools. The compression rates obtained by BdBG ranged from 0.2494 bpb (bits per base) in S.aureus to 1.2058 bpb in the gut of H.sapiens. Compared with GZIP, LEON, and MINCE, our proposed BdBG method achieved a satisfactory compression rate in all datasets. Our method improves upon the compression performances of GZIP, LEON, and MINCE by up to 83%, 81%, and 52%, respectively.

**Table 2:** Compression size for various datasets.

Dataset ID	Compression Rate (Bits Per Base)				BdBG File Size Reduction		
	GZIP	LEON	MINCE	BdBG	GZIP	LEON	MINCE
ERR1147042	2.4098	1.5757	0.8609	**0.5247**	−78.23%	−66.70%	−39.05%
ERR034088	2.3678	1.8970	1.1335	**0.9543**	−59.70%	−49.69%	−15.81%
SRR554369	2.4390	1.5541	0.7533	**0.5227**	−78.57%	−66.37%	−30.61%
SRR959239	1.6703	1.3978	0.3635	**0.2791**	−83.29%	−80.03%	−23.22%
ERR418881	0.4784	1.3152	0.5149	**0.2494**	−47.87%	−81.04%	−51.56%
MH0001.081026	2.5483	1.3176	1.3659	**1.2058**	−52.68%	−8.49%	−11.72%
SRR327342	2.4870	0.6655	0.6540	**0.6331**	−74.54%	−4.87%	−3.20%
SRR037452	2.5301	1.7720	1.0617	**0.9230**	−63.52%	−47.91%	−13.06%

## Discussion

We have shown that our method, BdBG, can achieve improved compression performance by combining the read bucketing and reference constructing methods. In the read sequence bucketing step, BdBG extends the MINCE (Patro & Kingsford, 2015) read sequence clustering method, which brings similar read sequences to close together and benefit the downstream compression. In the reference constructing step, The de Bruijn graphs we build continues the entire process of encoding the data, rather than constructing the entire de Bruijn graph at the beginning of the encoding, as in the LEON (Benoit et al., 2015) method does. Consequently, our method can avoid storing space-intensive de Bruijn graph, which can effectively improve the compression rate. Furthermore, BdBG makes full use of reads that have been encoded and improves the strategies for encoding the bifurcation file by the arithmetic encoder.

The results indicated that our method of constructing dynamic de Bruijn graphs with already-encoded read sequences was effective for compressing the genome and transcriptome sequencing dataset. For instance, when two similar read sequences have only one different nucleotide, the information (read index, position, nucleotide) must be recorded for the general-purpose Lempel–Ziv (Ziv & Lempel, 1977) compression tool. However, for our method with the de Bruijn graph, we only need to record the information (position, nucleotide) without keeping track of the reference read index, because all the reference sequence information is included in the de Bruijn graph. In a better case, if the node is a Branch node caused by the different nucleotide, we only need to record the information (nucleotide), which considerably reduces the storage space required to record the variances in the read sequence.

## Conclusions

In this paper, we proposed BdBG, a new alignment-free and reference-free compression method, based on the concept of bucketing similar reads into the same bucket and compressing reads in each bucket separately via dynamic de Bruijn graphs. By using the bucket index as the read ‘anchor’, BdBG only needs to record a list of bifurcations for encoding the raw read sequences in the de Bruijn graph. Moreover, BdBG employs the dynamic de Bruijn graphs as the encoding reference sequence, thereby avoiding storage of the de Bruijn graph and effectively further reducing the storage space. The experimental results for the different datasets demonstrate the effectiveness of our proposed BdBG over state-of-the-art methods.

## Additional Information and Declarations

### Data Availability

The following information was supplied regarding data availability: DDBJ (https://www.ddbj.nig.ac.jp/index-e.html): ERR1147042, ERR034088, ERR418881; ELBM-EBI (https://www.ebi.ac.uk/): SRR554369, SRR959239, SRR327342, SRR037452; BGI (https://www.bgi.com/) MH0001.081026 (http://public.genomics.org.cn/BGI/gutmeta/High_quality_reads/MH0001/081026/MH0001_081026_clean.1.fq.gz; http://public.genomics.org.cn/BGI/gutmeta/High_quality_reads/MH0001/081026/MH0001_081026_clean.2.fq.gz).
